# Functional Evaluation of a Rare Variant c.516G>C (p.Trp172Cys) in the *GJB2* (Connexin 26) Gene Associated with Nonsyndromic Hearing Loss

**DOI:** 10.3390/biom11010061

**Published:** 2021-01-05

**Authors:** Ekaterina A. Maslova, Konstantin E. Orishchenko, Olga L. Posukh

**Affiliations:** 1 Federal Research Center Institute of Cytology and Genetics, Siberian Branch of the Russian Academy of Sciences, Novosibirsk 630090, Russia; maslova@bionet.nsc.ru (E.A.M.); keor@bionet.nsc.ru (K.E.O.); 2 Novosibirsk State University, Novosibirsk 630090, Russia

**Keywords:** hearing loss, *GJB2*, variant c.516G>C (p.Trp172Cys), Connexin 26, gap junction channels, transgenic HeLa cell lines, functional assay

## Abstract

Mutations in the *GJB2* gene encoding transmembrane protein connexin 26 (Cx26) are the most common cause for hearing loss worldwide. Cx26 plays a crucial role in the ionic and metabolic homeostasis in the inner ear, indispensable for normal hearing process. Different pathogenic mutations in the *GJB2* gene can affect all stages of the Cx26 life cycle and result in nonsyndromic autosomal recessive (DFNB1) or dominant (DFNA3) deafness and syndromes associating hearing loss with skin disorders. This study aims to elucidate the functional consequences of a rare *GJB2* variant c.516G>C (p.Trp172Cys) found with high frequency in deaf patients from indigenous populations of Southern Siberia (Russia). The substitution c.516G>C leads to the replacement of tryptophan at a conserved amino acid position 172 with cysteine (p.Trp172Cys) in the second extracellular loop of Cx26 protein. We analyzed the subcellular localization of mutant Cx26-p.Trp172Cys protein by immunocytochemistry and the hemichannels permeability by dye loading assay. The *GJB2* knockout HeLa cell line has been generated using CRISPR/Cas9 genome editing tool. Subsequently, the HeLa transgenic cell lines stably expressing different *GJB2* variants (wild type and mutations associated with hearing loss) were established based on knockout cells and used for comparative functional analysis. The impaired trafficking of mutant Cx26-p.Trp172Cys protein to the plasma membrane and reduced hemichannels permeability support the pathogenic effect of the c.516G>C (p.Trp172Cys) variant and its association with nonsyndromic hearing loss. Our data contribute to a better understanding of the role of mutations in the second extracellular loop of Cx26 protein in pathogenesis of deafness.

## 1. Introduction

Hearing loss (HL) is one of the most common sensory disorders, affecting one in 500–1000 newborns and approximately half of congenital HL cases have a genetic etiology [1]. Hereditary HL is extremely heterogeneous: over 140 different genes are causally implicated in nonsyndromic HL and about 400 different syndromes include HL as one of the clinical symptoms [2,3]. Mutations in gene *GJB2* (gap junction protein, beta-2, 13q12.11, MIM 121011) encoding transmembrane protein connexin 26 (Cx26) are the main cause for nonsyndromic autosomal recessive deafness 1A (DFNB1A, MIM 220290) in many populations [4], making *GJB2* gene testing essential for the establishment of genetic diagnosis of HL.

Cx26 molecules have the same topological structure as other members of the connexin family containing twenty-one proteins: four transmembrane domains (TM1-4), two extracellular loops (E1 and E2), a cytoplasmic loop (CL), and cytoplasmic N- and C-terminal domains (NT and CT) [5]. Six connexin molecules associate to form transmembrane hemichannels (connexons) which dock with hemichannels of adjacent cells forming gap junctions that are essential for the transport of ions and other low-molecular-weight components between cells. Connexons may also form functional hemichannels, particularly in pathological conditions, at the non-junctional areas of the plasma membrane providing connection between the cell cytoplasm and the extracellular space [6,7].

Gap junctions are one of the key structures involved in cell-to-cell communication and many tissues and cell types express two or more members of the connexin family. Within the organ of Corti, Cx26 is synthesized by all supporting cell types, and plays a crucial role for the ionic and metabolic homeostasis in the inner ear indispensable for normal hearing process [8,9,10,11,12]. Additionally, Cx26 gap junctions are important for keratinocyte growth and differentiation in the skin [13,14,15].

Different pathogenic mutations in the *GJB2* gene can affect all stages of the Cx26 life cycle: biosynthesis, post-translational modifications, and degradation of Cx26 molecules, their oligomerization into connexons, intracellular trafficking of connexons to the plasma membrane, normal docking of the connexons from neighboring cells to form gap junction channels, and interactions between other co-expressed connexins [4,16,17,18,19]. The majority of *GJB2* mutations are recessively inherited and, being in homozygous or compound heterozygous states, result in nonsyndromic recessive deafness (DFNB1) while some of *GJB2* mutations are dominantly inherited and lead to nonsyndromic autosomal dominant deafness (DFNA3) or several different syndromes associating hearing loss with skin disorders [2,4,20,21,22,23,24,25].

Over 400 deafness-associated, as well as not yet well-classified, variations with ambiguous functional significance are described in the *GJB2* gene sequence (the Human Gene Mutation Database: http://www.hgmd.cf.ac.uk [26]). Numerous in vitro studies, using different expression models and a number of functional assays, have been performed for uncovering the disease-associated mechanisms of several dozen Cx26 mutants potentially involved in hearing loss [10,24,25,27,28,29,30,31,32,33,34,35,36,37,38,39,40,41,42,43,44,45,46,47,48].

In our previous studies, we evaluated the spectrum and frequency of the *GJB2* gene variants in a large cohort of deaf Tuvinian and Altaian patients and in the ethnically matched controls (Southern Siberia, Russia) [49,50,51]. Tuvinians and Altaians are the indigenous Turkic-speaking populations of two federal subjects of the Russian Federation, the Tyva Republic (Tuva) and the Altai Republic, located in Southern Siberia. Several known pathogenic and benign *GJB2* variants have been detected in examined patients. A striking finding was a high prevalence of a rare specific *GJB2* variant c.516G>C (p.Trp172Cys) identified in homozygous or compound heterozygous states in deaf Tuvinian patients [50]. Besides Tuvinians, c.516G>C was only found with less frequency in Altaians [49,51], in one deaf patient from Mongolia [52], and nowhere else in the world. A crucial role of the founder effect in high prevalence of c.516G>C (p.Trp172Cys) in indigenous populations of Southern Siberia has been established in our recent study [53]. The c.516G>C substitution leads to a replacement of an aromatic non-polar tryptophan with a small polar cysteine at conserved amino acid position 172 (p.Trp172Cys) in the second extracellular loop of protein connexin 26 (Cx26). The c.516G>C meets the main criteria to be classified as pathogenic for autosomal recessive hearing loss based on the ACMG/AMP criteria [54] as specified by the Hearing Loss Expert Panel [55]. However, the consequences of this variant on the Cx26 protein structure and function of the Cx26-channels remain unknown.

This study aims to perform the functional assessment of pathogenicity of a rare recessive *GJB2* variant c.516G>C (p.Trp172Cys) found in association with nonsyndromic deafness in indigenous peoples (Tuvinians and Altaians) of Southern Siberia (Russia). For comparative in vitro analysis of the functional effect of c.516G>C (p.Trp172Cys), we generated the panel of transgenic human cell lines with a stable expression of different *GJB2* variants (wild type and mutants associated with hearing loss).

## 2. Materials and Methods

### 2.1. GJB2 Cloning Strategy

The *GJB2* gene (NM_004004.6) coding region was amplified from the genomic DNA derived from previously examined deaf patients [49,50] carrying specific mutant alleles (c.516G>C, c.224G>A, c.35delG) and wild type allele (wt) by standard polymerase chain reaction (PCR) using primers Left_SfiI 5′-CTGTGTGGCCTCTGAGGCCCTTTTCCAGAGCAAACCGCC-3′ and Right_SfiI 5′-ATGCTGGGCCTGACAGGCCCTAACAACTGGGCAATGCGT-3′. The amplified products were cloned into the *SfiI* site of the pSBbi-GP vector, which was a gift from Eric Kowarz (Addgene plasmid #60511; http://n2t.net/addgene:60511; RRID: Addgene_60511). All constructs were sequenced to verify that no mutations were incorporated during PCR.

### 2.2. Cell Culture and Generating Cell Lines Expressing WT Cx26 or Cx26 Mutants

Cells were cultured in Dulbecco’s minimum essential medium supplemented with 10% fetal bovine serum, 100 U/mL penicillin, and 100 µg/mL streptomycin in a humidified atmosphere of 5% CO_2_ at 37 °C.

HeLa cell line was obtained from the cell collection of the State Research Center of Virology and Biotechnology “Vector” (Novosibirsk, Russia). HeLa cell line with a knockout of the *GJB2* gene (HeLa Cx26-KO) was generated using CRISPR/Cas9 genome editing tool. To introduce double-strand breaks flanking the *GJB2* coding region, two guide RNAs (gRNAs) were designed by Benchling online tools (https://benchling.com). The gRNA’s sequences were cloned into the pX330-U6-Chimeric_BB-CBh-hSpCas9 vector digested with *BbsI*. The pX330-U6-Chimeric_BB-CBh-hSpCas9 was a gift from Feng Zhang (Addgene plasmid #42230; http://n2t.net/addgene:42230; RRID: Addgene_42230). These two plasmids were then co-transfected with the pEGFP-N1 (Clontech Laboratories Inc., Mountain View, CA, USA) in a ratio of 5:1 into HeLa cells with ~ 70% confluency using Lipofectamine 3000. The EGFP fluorescence was monitored for transfection efficiency and used for cell sorting. After the transfection, 2000 cells were sorted on BD FACSAria™III Sorter by EGFP signal into a 10-cm cell culture dish. Subsequently, single colonies were picked and transferred using CellCelector™ system to a 24-well plate. Cell colonies were expanded and screened for the deletion of *GJB2* coding region by PCR and verified by sequencing. The breakpoints of the deletion were located in exon 2 of the *GJB2* gene (NG_008358.1) from 8336 bp to 9211 bp. The size of the deletion was 826 bp.

The Sleeping Beauty transposon system was used to generate cell lines expressing wt Cx26 or Cx26 mutants [56]. The pCMV(CAT)T7-SB100 transposase vector was co-transfected with the pSBbi-GP plasmid with one of specific variants of the *GJB2* coding region (wt, c.516G>C, c.224G>A, or c.35delG) in a ratio of 1:20 into HeLa Cx26-KO cells with ~ 70% confluency using Lipofectamine 3000. The pCMV(CAT)T7-SB100 plasmid was a gift from Zsuzsanna Izsvak (Addgene plasmid #34879; http://n2t.net/addgene:34879; RRID:Addgene_34879). Transfected cells were then selected by puromycin 2 μg/mL for 14 days. Surviving cells were propagated and 2000 GFP-positive cells were sorted with a BD FACSAria™III Cell Sorter into a 10-cm cell culture dish. Subsequently, single colonies were picked and transferred by CellCelector™ to a 12-well plate and were expanded.

### 2.3. Immunostaining and Image Acquisition

All reagents were heated to 37 °C. The cells were seeded into 12-well plate on coverslips at a density of 60,000 cells/well. At 70–80%, confluency cells were washed with phosphate-buffered saline (PBS) and fixed with pre-warmed 4% paraformaldehyde (PFA) in PBS for 20 min at room temperature (RT). After washing twice with PBS for 5 min, coverslips with cells were permeabilized with 0.1% Triton X-100 solution in PBS for 20 min at RT, followed by nonspecific protein blocking with 2% bovine serum albumin (BSA) in PBS for 1 h at RT. Further, the detection of Cx26 protein was performed using polyclonal primary antibodies (#51-2800, Thermo Fisher Scientific, Rockford, IL, USA, 1:250) diluted in 2% BSA in PBS for 1 h at RT. After washing twice with 0.2% Tween 20 in PBS for 5 min, coverslips were incubated for 1 h at RT in dark with Alexa Fluor Plus 555 conjugated goat anti-rabbit IgG (#A32732, Thermo Fisher Scientific, Rockford, IL, USA, 4 µg/mL) in 0.2% Tween 20 in PBS. Before mounting the cells, coverslips were washed three times with 0.2% Tween 20 in PBS for 5 min and then placed over DAPI-containing mounting medium (ProLong™ Diamond Antifade Mountant with DAPI, P36962, Thermo Fisher Scientific, Rockford, IL, USA) to stain nuclei. Images were recorded on Zeiss LSM 510 META confocal microscope with 63× oil immersion objective in the Core Facility for Microscopy of Biologic Objects, SB RAS, Novosibirsk, Russia (regulation no. 3054).

### 2.4. Western Blot

Cells were harvested by trypsinization for 5 min at 37 °C and washed with PBS. The cells were pelleted by centrifugation at 300× *g* for 5 min at 4 °C and lysed on ice for 15 min in RIPA buffer (150 mM NaCl, 1% Triton X-100, 0.5% sodium deoxycholate, 0.1% SDS, 50 mM Tris-HCl, pH 8.0) supplemented with protease inhibitors (5 mM NaF (Sigma-Aldrich, St. Louis, MO, USA), PhosSTOP (Roche, St. Louis, MO, USA), 1x Inhibitor Cocktail cOmplete™ ULTRA Tablets (Roche, St. Louis, MO, USA)). The cells were ruptured by sonication and lysates were cleared by centrifugation at 14,000× *g* for 20 min at 4 °C. Cell lysates were normalized for total protein with BCA assay (Pierce™ BCA Protein Assay Kit, Thermo Fisher Scientific, Rockford, IL, USA). Cell lysates were diluted in 4x Laemmli protein sample buffer (Bio-Rad, Hercules, CA, USA) and incubated at 65 °C for 20 min to denature proteins. Samples containing the same amount of protein were run on 15% SDS-PAGE. Proteins were transferred onto polyvinylidene difluoride membranes (0.45 µm, Bio-Rad, Hercules, CA, USA) and blocked in 5% nonfat dry milk (Cell Signaling; Beverly, MA, USA) for 1 h at RT on shaker. The membranes were probed at 4 °C overnight with primary antibodies (Rabbit polyclonal anti-Connexin 26 antibodies, 1:250, #51-2800, Thermo Fisher Scientific, Rockford, IL, USA and mouse monoclonal α-Tubulin (DM1A), 1:1000, #3873, Cell Signaling; Beverly, MA, USA) diluted in 5% nonfat dry milk. After washing with TBST (150 mM NaCl, 0.05% Tween20, 10 mM Tris-HCl, pH 8.0), membranes were probed with horseradish peroxidase–conjugated antibodies (Anti-mouse IgG, HRP-linked Antibody, 1:1000, #7076 and Anti-rabbit IgG, HRP-linked Antibody, 1:1000, #7074, Cell Signaling; Beverly, MA, USA). Blots were imaged by SuperSignal™ West Pico PLUS Chemiluminescent Substrate (#34580, Thermo Fisher Scientific, Rockford, IL, USA). The original Western blot is presented in Figure S1.

### 2.5. Cx26-Hemichannels Permeability Assay

Cells grown to ~60% confluence in 12-well plates and engineered to express wt Cx26 or Cx26 mutants as described above, were incubated for 40 min at RT in 0.15 mmol/L Propidium Iodide (PI, MW = 668.39) (P4170, Sigma-Aldrich, St. Louis, MO, USA) solution, prepared in either HBSS without Ca^2+^ ions (#14175053, Thermo Fisher Scientific, Rockford, IL, USA) containing 10 mmol/L EGTA and 1 mmol/L MgCl_2_, or HBSS with Ca^2+^ ions (#14025050, Thermo Fisher Scientific, Rockford, IL, USA). Cells were imaged using Zeiss AxioObserver.Z1 fluorescence microscope to visualize GFP and PI. All images were captured with a 10x objective at RT. The PI-positive cells were counted manually. Total number of the cells was measured by automated recognition of the GFP-positive cells using the CascadeRCNN neural network software [57]. From 14 to 20 images were analyzed for each of examined cell lines (in total, ~8500–15,500 cells per line). Pearson’s chi-squared test with significance level of *p* < 0.05 was applied to compare the proportion of cells accumulated the PI dye. Data are expressed as mean ± standard error of the mean (SEM).

In addition, PI permeability of Cx26-hemichannels was analyzed by flow cytometry. Cells were grown and incubated in PI solution in HBSS with or without Ca^2+^ ions as described above. Cells were detached with trypsin/EDTA and washed three times with HBSS buffer with Ca^2+^ ions. After washing, cells were resuspended in growth medium and analyzed by BD FACSAria™III Cell Sorter in the Core Facility for Flow Cytometry at the Institute of Cytology and Genetics SB RAS (Novosibirsk, Russia).

## 3. Results

Our previous study revealed a rare missense variant c.516G>C in the *GJB2* gene leading to a replacement of tryptophan (Trp) with cysteine (Cys) at the 172 amino acid position of the Cx26 molecule (p.Trp172Cys). A distinct segregation of homozygosity or compound heterozygosity for c.516G>C (p.Trp172Cys) with nonsyndromic hearing loss in affected families and multiple in silico predictions suggest potential deleterious impact of c.516G>C (p.Trp172Cys) on the Cx26 structure and function [50].

To elucidate the effect of variant c.516G>C (p.Trp172Cys), we performed comparative functional analysis including the examination of subcellular localization of mutant Cx26 molecules by immunocytochemistry (ICC) and the hemichannels permeability assay by propidium iodide (PI) dye transfer.

### 3.1. Generation of GJB2-Transgenic HeLa Cell Lines

HeLa cell line was chosen as an experimental model. Since the current data on the level of endogenous *GJB2* (Cx26) expression in HeLa cell lines are ambiguous [40,58,59,60,61,62], we primarily established the *GJB2* knockout HeLa cells (HeLa Cx26-KO) with deletion of whole *GJB2* coding region for subsequent generation of transgenic cell lines to avoid any *GJB2* expression in the used HeLa cell line.

For comparative functional analysis of variant c.516G>C (p.Trp172Cys), the panel of transgenic HeLa cell lines stably expressing different *GJB2* coding region variants was generated using Sleeping Beauty transposon system: HeLa-p.W172C for c.516G>C (p.Trp172Cys), HeLa-p.R75Q for c.224G>A (p.Arg75Gln), HeLa-c.35delG for c.35delG (p.Gly12Valfs*2), and HeLa-Cx26wt for wild type (wt). The substitution c.224G>A leads to the replacement of arginine (Arg) with glutamine (Gln) at the 75th conserved amino acid position (p.Arg75Gln) at the junction between the first extracellular loop (EC1) and the second transmembrane domain (TM2) of Cx26. The p.Arg75Gln mutation has been reported to cause nonsyndromic autosomal dominant hearing loss (DFNB3) or hearing loss associated with palmoplantar keratoderma [34,45,49,63,64,65]. The c.35delG (deletion of one guanine in a sequence of six Gs at nucleotides 30–35) is common recessive *GJB2* mutation associated with nonsyndromic autosomal recessive hearing loss (DFNB1) [4,66]. This frameshift mutation is predicted to lead to a synthesis of truncated Cx26 protein (p.Gly12Valfs*2).

### 3.2. Functional Studies

Functional studies to determine cellular localization of mutant Cx26-p.W172C protein and to assess the Cx26-p.W172C hemichannels permeability in comparison with other Cx26 mutants and Cx26 wild type were carried out on the transgenic lines HeLa-p.W172C, HeLa-p.R75Q, HeLa-c.35delG, and HeLa-Cx26wt. The antibodies against 13 amino acids in C-terminal domain of Cx26 were used for the ICC visualization of the Cx26s and PI was used for the dye uploading assay.

#### 3.2.1. Cellular Localization of Cx26 Variants

**Figure 1:**
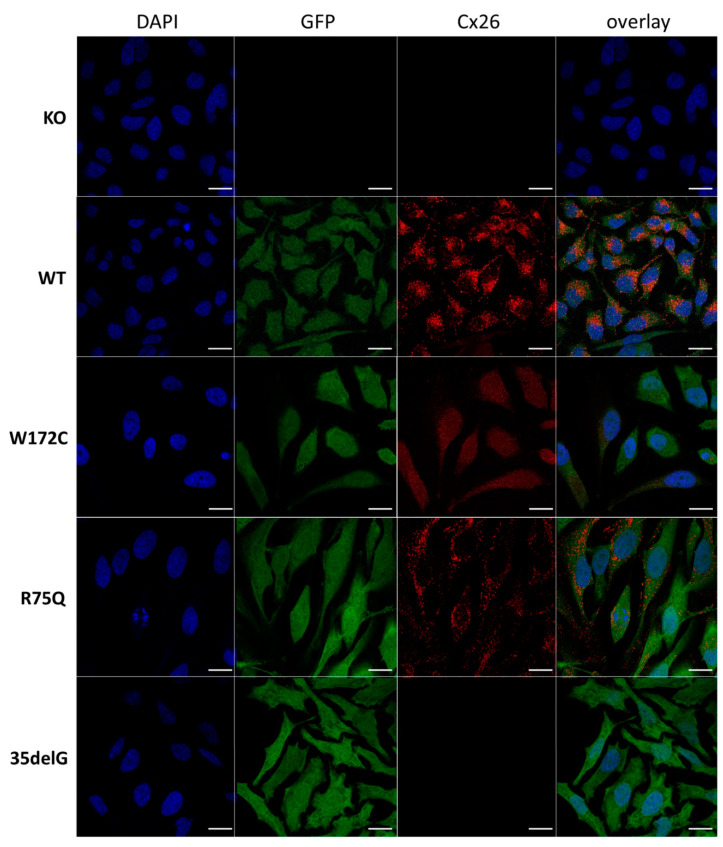
Localization of Cx26 in several newly established HeLa cell lines (confocal micrographs). The panels of Cx26 variants are designated (top down) as KO, WT, W172C, R75Q, and 35delG for cell lines HeLa Cx26-KO, HeLa-Cx26wt, HeLa-p.W172C, HeLa-p.R75Q, and HeLa-c.35delG, respectively. Nuclei were visualized with DAPI (blue), transgenic HeLa cells were visualized with Green Fluorescent Protein (GFP) and Cx26 was immunostained with antibodies against C-terminus of Cx26 (red). Scale bar = 20 µm.

We revealed different patterns of cellular localization of the Cx26 variants by ICC in analyzed transgenic HeLa lines (Figure 1).

**Figure 2:**
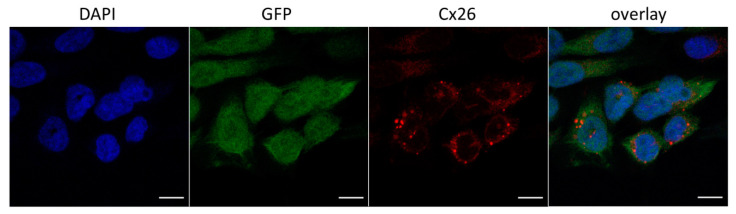
A fraction of HeLa-p.W172C cells with reduced number of protein granules (confocal micrographs). Blue color indicates DAPI nuclei staining, green color corresponds to GFP signal, red color corresponds to Cx26 signal. Scale bar = 10 μm.

No specific signal was detected in HeLa Cx26-KO confirmed the lack of Cx26 expression in this line. The Cx26 wild type and mutant Cx26-p.R75Q, both visible as distinct clusters of protein, were predominantly localized at the plasma membrane. The observed membrane localization of the Cx26-p.R75Q protein, similar to Cx26 wild type, is consistent with previous studies [25,34,41,45]. Mutant Cx26-p.W172C typically exhibited significantly smaller and less bright puncta diffusely distributed predominantly in the cell cytoplasm. However, along with such cells, Cx26-p.W172C–containing rare discrete protein granules were observed in some cells (Figure 2). The lack of a specific signal for HeLa-c.35delG was initially expected since the antibodies against 13 amino acids in Cx26 C-terminal domain were used (Figure 1).

**Figure 3:**
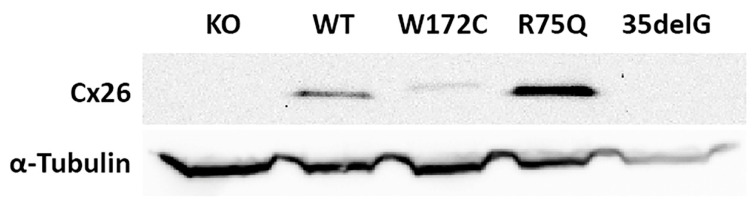
Analysis of the Cx26 protein expression in transgenic HeLa cell lines. The total lysates of cells were subjected to Western blot analysis with antibodies against Cx26 and the loading control, α-tubulin. The panels of Cx26 variants are designated as KO, WT, W172C, R75Q, and 35delG for cell lines HeLa Cx26-KO, HeLa-Cx26wt, HeLa-p.W172C, HeLa-p.R75Q, and HeLa-c.35delG, respectively.

Further, we have confirmed the presence of Cx26 in transgenic HeLa cell lines at protein level by Western blot analysis. Different patterns of the Cx26 expression, consistent with the ICC visualization of the Cx26s, were revealed in the investigated cell lines: the HeLa Cx26-KO and HeLa-c.35delG lines exhibited the expected absence of Cx26 protein while the expression of the p.W172C-mutant protein was reduced compared to other lines expressing Cx26 (HeLa-Cx26wt and HeLa-p.R75Q) (Figure 3).

#### 3.2.2. Evaluation of Cx26-Hemichannels Permeability by PI Dye Loading Assay

**Figure 4:**
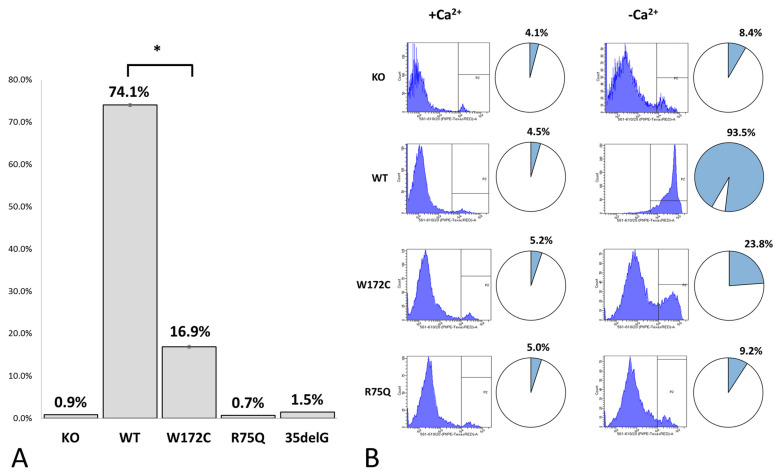
Percentage of cells loaded with propidium iodide (PI) through hemichannels. (**A**) Proportion of PI-positive cells estimated by analysis of fluorescent microscopy images (the data were obtained for cells in Ca^2+^-free HBSS). Bars represent the mean (± SEM) for each of analyzed cell lines. * denotes significant differences at *p* < 0.05. (**B**) Percentage of cells loaded with PI estimated by flow cytometry in Ca^2+^-containing (left) and Ca^2+^-free (right) extracellular conditions. KO, WT, W172C, R75Q, and 35delG denote lines HeLa Cx26-KO, HeLa-Cx26wt, HeLa-p.W172C, HeLa-p.R75Q, and HeLa-c.35delG, respectively.

The analysis of Cx26-hemichannels permeability in examined cell lines with different Cx26 variants was performed by PI dye loading assay both in Ca^2+^-containing and Ca^2+^-free extracellular conditions. The calcium-dependent gating mechanism of Cx-hemichannels is well explored: high extracellular concentration of Ca^2+^ keeps Cx26-hemichannels in closed state whereas low concentration or lack of Ca^2+^ stimulates hemichannels to open [67,68]. The permeability of Cx26-hemichannels was estimated as a proportion of PI-positive cells detected on the fluorescent microscope images (Figure 4A) or by flow cytometry (Figure 4B). The representative fluorescent microscopy images of the PI-loaded cells in different cell lines are presented in Figure S2. Only 0.9%, 0.7%, and 1.5% of cells were PI-positive in lines HeLa Cx26-KO, HeLa-p.R75Q, and HeLa-c.35delG, respectively. At the same time, 74.1% of cells in HeLa-Cx26wt and 16.9% of cells in HeLa-p.W172C exhibited PI loading (Figure 4A). In HeLa-p.W172C cells the PI uptake was significantly weaker compared to HeLa-Cx26wt (*p* < 0.05) (Figure 4A). Similar results were obtained by flow cytometry (Figure 4B). Since PI is a marker commonly used in fluorescence microscopy and flow cytometry for dead cells with compromised membrane integrity, a small proportion of PI-positive cells detected in HeLa Cx26-KO line, as well as in other cell lines, can be attributed to dead cells usually present in cell cultures (Figure 4A,B).

Our results suggest that the variant c.516G>C in the *GJB2* gene affects the intracellular localization of the Cx26 protein and significantly reduces hemichannels permeability.

## 4. Discussion

Our previous study revealed a rare missense variant c.516G>C (p.Trp172Cys) in the *GJB2* gene associated with nonsyndromic recessive hearing loss in indigenous populations in Southern Siberia (Russia) [49,50,51]. Several lines of evidence (segregation with hearing loss, rarity, in silico predictions) support pathogenicity of c.516G>C (p.Trp172Cys), however the functional consequences of this variant on the structure and function of the Cx26 protein remain unknown. In order to elucidate the pathogenicity of c.516G>C (p.Trp172Cys), we performed functional studies regarding the ability of mutant Cx26-p.W172C protein to traffic to the cell membrane as well as its effect on functionality (permeability) of hemichannels.

**Figure 5:**
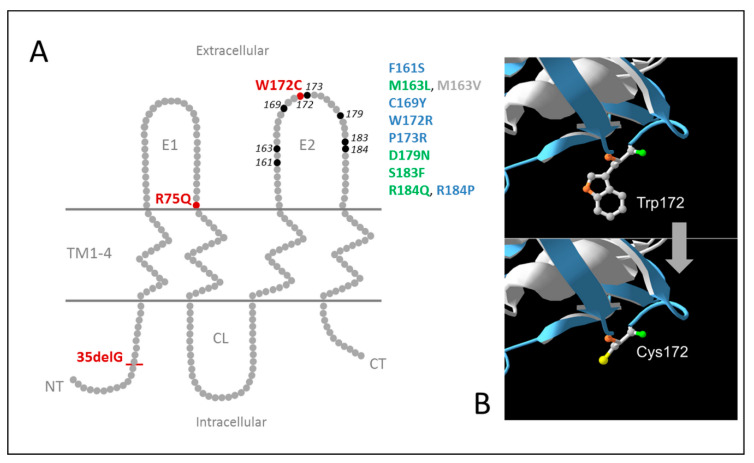
(**A**) Topological structure of Connexin 26. TM1-4—four transmembrane domains, E1 and E2—extracellular loops, CL—cytoplasmic loop, NT—N-terminal segment, CT—C-terminal segment. The variants p.Trp172Cys (W172C), p.Arg75Gln (R75Q), p.Gly12Valfs*2 (35delG) examined in this study are shown in red. Amino acid positions for ”pathogenic” and ”likely pathogenic” *GJB2* variants located in E2 previously subjected to functional analysis [19,25,27,28,38,40,41,43,46,48,69,70] are marked in black. The mutations linked to recessive deafness are marked in blue; the mutations linked to dominant hearing loss (nonsyndromic and syndromic) are marked in green; ambiguously defined (recessive or dominant) mutation p.M163V is shown in grey. (**B**) Wild (Trp172) and mutant (Cys172) types of Cx26 (modified Figure 1 from [50]).

The panel of transgenic human cell lines with a stable expression of different *GJB2* variants (wild type and mutants associated with hearing loss) was generated for in vitro analysis of functional effect of c.516G>C (p.Trp172Cys). For comparative analysis, along with the *GJB2* wild type, two well-characterized *GJB2* mutations: recessive c.35delG (p.Gly12Valfs*2) leading to the synthesis of truncated Cx26 protein and dominant c.224G>A (p.Arg75Gln) causing DFNB3 or hearing loss associated with palmoplantar keratoderma, were chosen as controls (Figure 5A).

Cellular localization and hemichannels permeability were investigated by the immunocytochemistry and the PI dye uptake assay. The trafficking of mutant Cx26-p.W172C to the cell membrane in most of HeLa-p.W172C cells was apparently impaired compared to wild type Cx26 and mutant Cx26-p.R75Q proteins (Figure 1). In addition, a reduced amount of mutant Cx26-p.W172C protein was detected by Western blot analysis in HeLa-p.W172C cells. However, along the cells exhibited an intracellular diffuse distribution of the Cx26-p.W172C protein, there was a small fraction of cells containing rare discrete protein granules (Figure 2). The PI uptake assay revealed significantly reduced hemichannel permeability (estimated as proportion of PI-positive cells) in HeLa-p.W172C line compared to HeLa-Cx26wt (Figure 4). Apparently, the PI-positive cells in HeLa-p.W172C line can represent the cells with partly functional hemichannels formed by mutant Cx26-p.W172C molecules. Based on all data obtained, we speculate that the impaired effect of variant p.Trp172Cys may be due to premature degradation of mutant Cx26-p.W172C molecules and their reduced capability of oligomerization.

Cx26 molecules have the same topological structure as other members of the Cx-protein family: four transmembrane domains (TM1–4), two extracellular loops (E1 and E2), a cytoplasmic loop (CL), and cytoplasmic N- and C-terminal domains. Six connexin molecules associate to form transmembrane hemichannels (connexons). The flux system through the gap junctions includes positively charged cytoplasmic entrance, a funnel, a negatively charged path, and an extracellular space [5]. Mutations in the *GJB2* gene linked to deafness have been found in all Cx26 domains and they can compromise different stages of the Cx26 life cycle. Along with obviously pathogenic *GJB2* variants severely truncating the Cx26 protein, a significant portion of deafness-associated *GJB2* variants is represented by various single amino acid residue changes located in different regions of the Cx26 protein sequence.

Many studies aimed to highlight the specific roles of each domain of Cx26 protein reviewing the pathogenic effects of different mutations associated with nonsyndromic or syndromic hearing impairments in *GJB2* gene and, as the different members of the connexin protein family are very similar in their topological organization, in other connexin genes [4,17,28,44,71,72,73,74,75,76,77].

Xu and Nicholson (2013) analyzed the distribution of the reported *GJB2* mutations associated with nonsyndromic hearing loss and revealed that TM2 domain of Cx26 shows the highest density of them (67%), followed by TM3 (50%), E2 (43%), TM4 (40%), NT, and CL (36%), TM1 and E1 (33%). When the frequency of mutagenesis leading to all forms of deafness, including syndromic forms, is considered, 5 domains show higher than 50% mutation rates (NT, TM2, TM3, E1 and E2) [74]. Although TM4 tends to be less important for the gap junction functioning [74], the mutations in TM4 can cause a range of phenotypes of dysfunctional gap junctions [44]. The cluster of deafness-causing mutations located at the border of the TM1 and E1 domains has been reported [74]. It shares the localization with Ca^2+^ coordinating amino acid residues identified by Bennet et al. [76]. Bai et al. (2018) suggested that the first extracellular loop (E1) is less important for docking compatibility; rather, the second extracellular loop (E2) serves this function [77].

The c.516G>C substitution analyzed in this study, leads to a replacement of an aromatic non-polar tryptophan with a small polar cysteine at conserved amino acid position 172 (p.Trp172Cys) in the second extracellular loop (E2) of Cx26 (Figure 5).

To elucidate the potential role of the second extracellular loop of Cx26 in hearing loss due to the mutations spectrum located in this domain, we summarized the missense variants located in E2 and defined as ”pathogenic” and ”likely pathogenic” in the Deafness Variation Database (http://deafnessvariationdatabase.org/) [78]. These mutations result in nonsyndromic recessive or dominant deafness and hearing loss associated with skin disease (Table S1). Interestingly, some mutations leading to hearing impairment are caused by different amino acid residue substitutions occurring at the same position.

Although about 30 different ”pathogenic” and ”likely pathogenic” missense variants have been reported in E2, the functional consequences have been examined only for a fraction of them [19,25,27,28,38,40,41,43,46,48,69,70] (Table S1, Figure 5A). Ten mutations were analyzed in these functional studies: dominant mutations (nonsyndromic p.M163L, p.D179N, p.R184Q, and syndromic p.S183F associated with deafness and focal palmoplantar keratoderma), mutations linked to nonsyndromic recessive deafness (p.F161S, p.C169Y, p.W172R, p.P173R, and p.R184P), and an ambiguously defined (recessive or dominant) mutation p.M163V (Figure 5A). Different model cell lines and experimental platforms (the transfected HeLa, HEK-293, NEB1 cells or the cochlear-relevant HEI-OC1 cells from mice, and the paired Xenopus oocytes expression system) were used for the analysis of expression, cellular localization and membrane trafficking of the mutant proteins, their ability to form functional gap junctions, and the relationship with the Cx26 wild type and other expressed connexins (Cx30, Cx43).

In a study of dominant mutation p.S183F causing a syndrome of focal palmoplantar keratoderma with deafness, de Zwart-Storm et al. (2008) have shown that p.S183F leads to a partial trafficking defect with the mutant protein accumulating in the cytoplasm, but retaining its ability to form gap junction plaques with residual channel activity [69]. Press et al. (2017) revealed that S183F mutant has defects in connexin trafficking as well as impaired gap junction and hemichannel function and exhibited transdominant effects on co-expressed wild type Cx26, Cx30, and Cx43 [48]. Consistent with previous studies of this mutation, Beach et al. [19] reported that the dominant syndromic S183F mutant was able to traffic to the plasma membrane (although some intracellular reservoirs of S183F were found) and to form gap junctions incapable of transferring a dye [48,70]. In addition, the S183F mutant co-localized in the same gap junctions as wild type Cx26 and Cx30, and gained a capacity to intermix with Cx43 within gap junctions [70]. The functional consequences of mutations p.M163L, p.D179N, and p.R184Q linked to nonsyndromic dominant deafness (DFNB3) have been investigated in four studies [25,38,41,43]. Matos et al. (2008) revealed that the trafficking of the mutant M163L-Cx26 to the cell membrane was clearly impaired; however, the trafficking to cell membrane with formation of gap junction-like structures was observed when this mutant was co-expressed with either wtCx26 or wtCx30. In addition, the expression of M163L-Cx26 caused an increased cell death compared to wtCx26 [38]. Su et al. (2010) found the accumulation of the R184Q mutant in the Golgi apparatus rather than in the cytoplasmic membrane and proposed the dominant-negative effect of p.R184Q on the function of WT Cx26 and Cx30 when Cx26R184Q co-expressed with either Cx26WT or Cx30WT [43]. Yum et al. (2010) and Zhang et al. (2011) have also demonstrated the dominant-negative effects of p.R184Q, as well as p.D179N, on Cx26 and Cx30 [25,41]. By functional analysis of mutation p.M163V causing nonsyndromic hearing loss, Press et al. (2017) have shown that the M163V mutant exhibited impaired gap junction function consistent with the study by Bruzzone et al. [28], with no transdominant properties associated with other co-expressed members of the connexin family [48].

Thus, dominant *GJB2* mutations in the second extracellular loop of Cx26 are mostly manifested as “gain of function” mutations with a transdominant effects on other co-expressed connexins whereas recessive mutations in E2 (reviewed below) closely match “loss of function” mutations.

Thönnissen et al. (2002) investigated functional implications of nonsyndromic recessive mutations p.F161S, p.P173R, and p.R184P regarding the protein stability, the assembly, and the gap junction-mediated intercellular communication [27]. Immunofluorescence analysis showed very weak membrane localization for F161S and no signal corresponding to P173R and R184P. The F161S, P173R, and R184P mutants did not reveal intercellular coupling in tracer coupling experiments, and the oligomerization studies demonstrated a complete absence of hemichannel formation in the R184P mutant, and only as monomeric protein [27]. Mani et al. (2009) described defective localization of the R184P mutant protein found largely in the cytoplasm [40]. The results of Bruzzone et al. (2003) in the paired Xenopus oocytes expression system, as well as of Beach et al. (2020) in the cochlear-relevant HEI-OC1 cells, supported the R184P inability to form intercellular channels [19,28]. The recessive mutation p.C169Y previously classified as a polymorphism, has been identified as causative of severe hearing loss in two Qatari families. That was supported by a functional study and molecular dynamics simulations: the C169Y mutant protein failed to form junction channels in HeLa transfectants despite being correctly targeted to the plasma membrane due to the disruption of the disulfide bridge that Cys169 forms with Cys64 in the wild type structure (Cx26WT) [46].

The replacement of tryptophan with arginine at position 172 (p.Trp172Arg) caused by substitution c.514T>A, has been described in the study by Mani et al. (2009) where two deaf patients from India were found to be homozygous for p.Trp172Arg [40]. To our knowledge, the p.Trp172Arg is the only other pathogenic missense variant at the same amino acid position (Trp172) as the p.Trp172Cys (c.516G>C) variant analyzed in our study. Mani et al. have shown that Trp172Arg-mutated Cx26 exhibited membrane localization similar to wild type Cx26, however, no dye (Lucifer yellow) transfer was observed for Trp172Arg-mutated Cx26 suggesting defective gap junction activity. Both extracellular loops (E1 and E2) of Cx26 molecule contain six conserved cysteines (C53, C60, C64 in E1 and C169, C174, C180 in E2) that form intramolecular disulfide bonds, playing an essential role in Cx26 hemichannel subunits docking [5]. Mani et al. suggested that replacement of tryptophan 172, located in close proximity to at least two of three cysteine residues in E2, could result in defective docking of two opposing connexon hemichannels [40]. Data on the impaired activity of Cx26-p.W172C-hemichannels obtained in our study are consistent with the study by Mani et al. However, abnormal cellular localization of mutant Cx26-p.W172C protein, observed in our study, may indicate a more pronounced effect of replacing Trp172 by Cys (p.Trp172Cys) than by Arg (p.Trp172Arg). Intramolecular structural changes due to the appearance of a new cysteine in the E2 of the Cx26 molecule can lead to impaired conformation, stability, and ability of oligomerization of the mutant Cx26-p.W172C protein.

## 5. Conclusions

In conclusion, we presented some functional evidences supporting the pathogenicity of the rare missense variant c.516G>C (p.Trp172Cys) in the *GJB2* gene associated with nonsyndromic recessive hearing loss (DFNB1). Our data contribute to a better understanding of the role of mutations in the second extracellular loop of Cx26 protein in pathogenesis of hearing loss. However, further studies are required to clarify the mechanisms of presumed effects of the p.Trp172Cys mutation on three-dimensional (3D) structure, cellular trafficking, stability, oligomerization, and life cycle of the mutant Cx26-p.Trp172Cys protein.

## Data Availability

The data presented in this study are available in this article and supplementary materials.

## Supplementary Materials

The following are available online at https://www.mdpi.com/2218-273X/11/1/61/s1, Figure S1: The results of Western blotting analysis (original Western blotting for Figure 3), Figure S2: PI loading through Cx26-hemichannels in examined HeLa cell lines (fluorescent microscopy). KO, WT, W172C, R75Q, and 35delG denote lines HeLa Cx26-KO, HeLa-Cx26wt, HeLa-p.W172C, HeLa-p.R75Q, and HeLa-c.35delG, respectively. Green color corresponds to GFP signal, red color corresponds to PI signal. Scale bar = 200 μm, Table S1: Missense variants of the *GJB2* gene in the extracellular loop 2 (E2) of Connexin 26 defined as ‘pathogenic’ and ‘likely pathogenic’ in the Deafness Variation Database (http://deafnessvariationdatabase.org/).
