# Supplementary material for: Functional Evaluation of a Rare Variant c.516G>C (p.Trp172Cys) in the GJB2 (Connexin 26) Gene Associated with Nonsyndromic Hearing Loss
Source: Biomolecules. 2021 Jan 5;11(1):61. doi: 10.3390/biom11010061 (PMC7824951; doi:10.3390/biom11010061)
Supplement: Supplementary file 1 [file biomolecules-11-00061-s001.pdf]

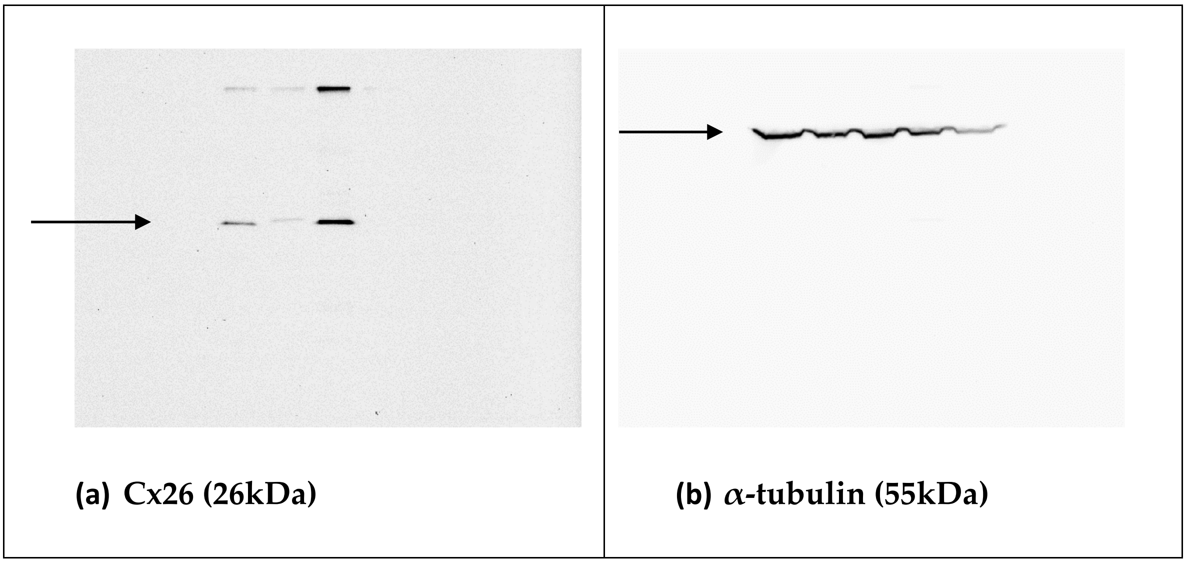

Figure S1. The results of Western blotting analysis (original Western blotting for Figure 3). Representative immunoblotting for: (a) Cx26 and (b) α-tubulin.

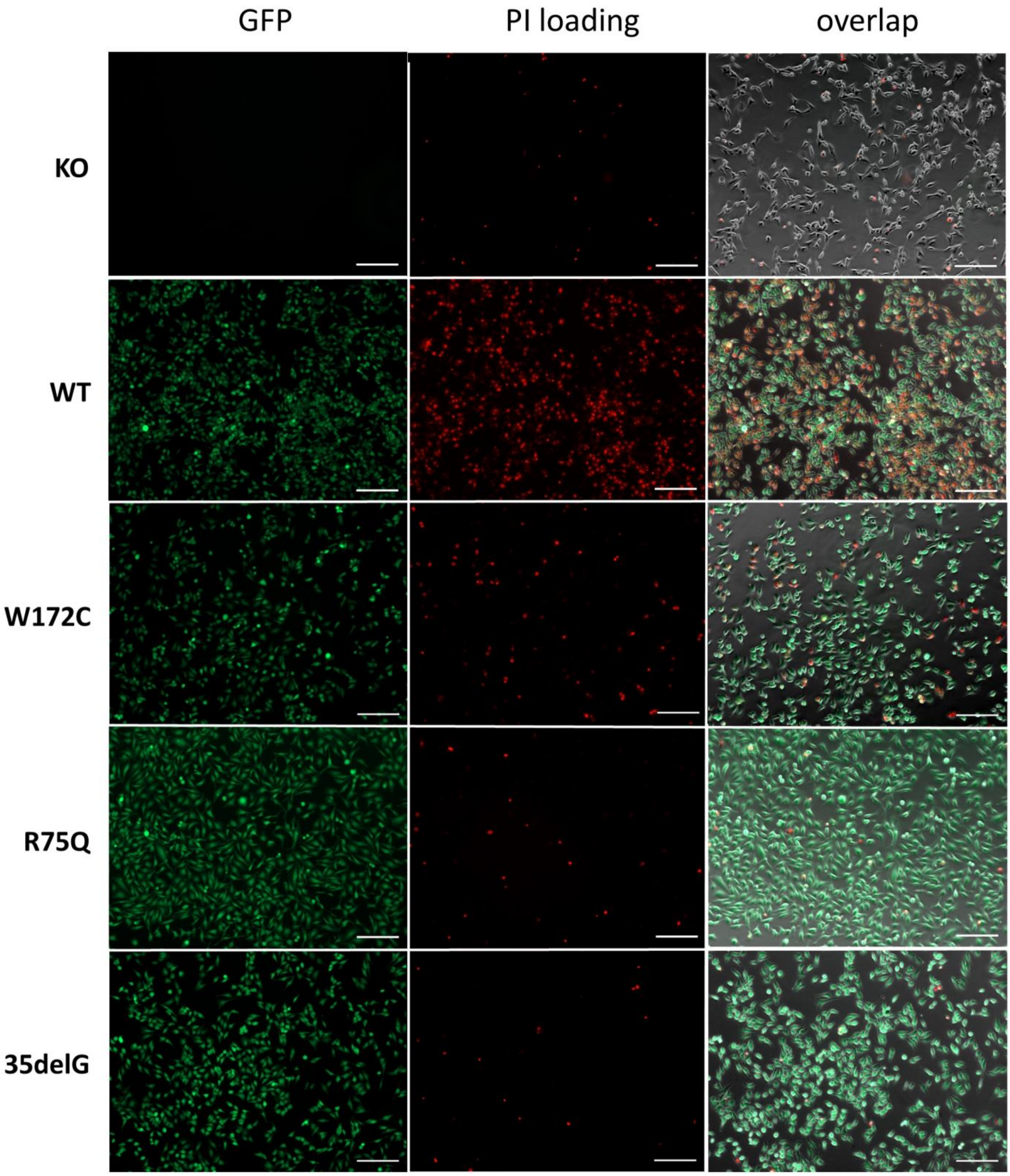

Figure S2. PI loading through Cx26-hemichannels in examined HeLa cell lines (fluorescent microscopy). KO, WT, W172C, R75Q, and 35delG denote lines HeLa Cx26-KO, HeLa-Cx26wt, HeLa-p.W172C, HeLa-p.R75Q, and HeLa-c.35delG, respectively. Green color corresponds to GFP signal, red color corresponds to PI signal. Scale bar = 200 μm.

Table S1. Missense variants of the *GJB2* gene in the extracellular loop 2 (E2) of Connexin 26 defined as 'pathogenic' and 'likely pathogenic' in the Deafness Variation Database (http://deafnessvariationdatabase.org/).

Amino acid position	Nucleotide change	Missense mutation	Pathogenic / Likely pathogenic	Phenotype	Functional studies
**158**	c.473A>G	**p.Tyr158Cys**	pathogenic	Sensorineural hearing loss	
**159**	c.475G>T	**p.Asp159Tyr**	likely pathogenic	Deafness	
	c.476A>T	**p.Asp159Val**	pathogenic	Deafness	
	c.475G>A	**p.Asp159Asn**	likely pathogenic	Deafness	
**161**	c.482T>C	**p.Phe161Ser**	pathogenic	Deafness	Thönnissen et al., 2002
**163**	c.487A>C	**p.Met163Leu**	pathogenic	Deafness, autosomal dominant 3	Matos et al., 2008
	c.487A>G	**p.Met163Val**	pathogenic	Deafness	Brussone et al., 2003; Press et al., 2017
	c.488T>C	**p.Met163Thr**	pathogenic	Sensorineural hearing loss	
**169**	c.505T>C	**p.Cys169Arg**	likely pathogenic	Hearing loss, non-syndromic	
	c.506G>A	**p.Cys169Tyr**	pathogenic	Deafness	Zonta et al., 2015
**170**	c.509A>C	**p.Asn170Thr**	likely pathogenic	Sensorineural hearing loss	
**171**	c.511G>T	**p.Ala171Ser**	likely pathogenic	Sensorineural hearing loss	
**172**	c.514T>A	**p.Trp172Arg**	pathogenic	Deafness	Mani et al., 2009
	c.516G>C	**p.Trp172Cys**	pathogenic	Deafness, nonsyndromic sensorineural	**this study**
**173**	c.517C>T	**p.Pro173Ser**	pathogenic	Deafness, autosomal recessive 1	
	c.518C>G	**p.Pro173Arg**	pathogenic	Deafness, autosomal recessive 1	Thönnissen et al., 2002
**174**	c.520T>C	**p.Cys174Arg**	pathogenic	Deafness, autosomal recessive 1	
	c.521G>C	**p.Cys174Ser**	likely pathogenic	Hearing loss	
**175**	c.523C>A	**p.Pro175Thr**	pathogenic	Deafness, autosomal recessive 1	
**178**	c.533T>C	**p.Val178Ala**	pathogenic	Deafness, autosomal recessive 1	
**179**	c.535G>A	**p.Asp179Asn**	pathogenic	Deafness	Yum et al., 2010; Zhang et al., 2011
	c.535G>C	**p.Asp179His**	likely pathogenic	Sensorineural hearing loss	
**183**	c.548C>T	**p.Ser183Phe**	pathogenic	Focal palmoplantar keratoderma with Deafness	de Zwart-Storm et al., 2008; Shuja et al, 2016; Press et al., 2017; Beach et al., 2020
**184**	c.550C>G	**p.Arg184Gly**	pathogenic	Deafness	
	c.550C>T	**p.Arg184Trp**	pathogenic	Deafness, autosomal recessive 1	
	c.551G>A	**p.Arg184Gln**	pathogenic	Deafness, autosomal dominant 3	Su et al., 2010; Yum et al., 2010; Zhang et al., 2011
	c.551G>C	**p.Arg184Pro**	pathogenic	Deafness, autosomal recessive 1	Thönnissen et al., 2002; Brussone et al., 2003;

					Mani et al., 2009; Beach et al., 2020
**186**	c.557C>A	**p.Thr186Lys**	pathogenic	Sensorineural hearing loss	
**188**	c.563A>G	**p.Lys188Arg**	pathogenic	Sensorineural hearing loss	
**190**	c.569T>A	**p.Val190Asp**	pathogenic	Deafness, nonsyndromic	

References for Table S1

Beach, R.; Abitbol, J.M.; Allman, B.L.; Esseltine, J.L.; Shao, Q.; Laird, D.W. *GJB2* Mutations Linked to Hearing Loss Exhibit Differential Trafficking and Functional Defects as Revealed in Cochlear-Relevant Cells. *Front Cell Dev Biol.* **2020**, *8*, 215.

Bruzzone, R.; Veronesi, V.; Gomès, D.; Bicego, M.; Duval, N.; Marlin, S.; Petit, C.; D'Andrea, P.; White, T.W. Loss-of-function and residual channel activity of connexin26 mutations associated with non-syndromic deafness. *FEBS Lett.* **2003**, *533*, 79–88.

de Zwart-Storm, E.A.; van Geel, M.; van Neer, P.A.; Steijlen, P.M.; Martin, P.E.; van Steensel, M.A. A novel missense mutation in the second extracellular domain of *GJB2*, p.Ser183Phe, causes a syndrome of focal palmoplantar keratoderma with deafness. *Am J Pathol.* **2008**, *173*, 1113–1119.

Mani, R.S.; Ganapathy, A.; Jalvi, R.; Srikumari Srisailapathy, C.R.; Malhotra, V.; Chadha, S.; Agarwal, A.; Ramesh, A.; Rangasayee, R.R.; Anand, A. Functional consequences of novel connexin 26 mutations associated with hereditary hearing loss. *Eur. J. Hum. Genet.* **2009**, *17*, 502–509.

Matos, T.D.; Caria, H.; Simões-Teixeira, H.; Aasen, T.; Dias, O.; Andrea, M.; Kelsell, D.P.; Fialho, G. A novel M163L mutation in connexin 26 causing cell death and associated with autosomal dominant hearing loss. *Hear Res.* **2008**, *240*, 87–92.

Press, E.R.; Shao, Q.; Kelly, J.J.; Chin, K.; Alaga, A.; Laird, D.W. Induction of cell death and gain-of-function properties of connexin26 mutants predict severity of skin disorders and hearing loss. *J Biol Chem.* **2017**, *292*, 9721–9732.

Shuja, Z.; Li, L.; Gupta, S.; Meşe, G.; White, T.W. Connexin26 Mutations Causing Palmoplantar Keratoderma and Deafness Interact with Connexin43, Modifying Gap Junction and Hemichannel Properties. *J Invest Dermatol.* **2016**, *136*, 225–235.

Su, C.C.; Li, S.Y.; Su, M.C.; Chen, W.C.; Yang, J.J. Mutation R184Q of connexin 26 in hearing loss patients has a dominant-negative effect on connexin 26 and connexin 30. *Eur J Hum Genet.* **2010**, *18*, 1061–1064.

Thönnissen, E.; Rabionet, R.; Arbonès, M.L.; Estivill, X.; Willecke, K.; Ott, T. Human connexin26 (*GJB2*) deafness mutations affect the function of gap junction channels at different levels of protein expression. *Hum Genet.* **2002**, *111*, 190–197.

Yum, S.W.; Zhang, J.; Scherer, S.S. Dominant connexin26 mutants associated with human hearing loss have trans-dominant effects on connexin30. *Neurobiol Dis.* **2010**, *38*, 226–236.

Zhang, J.; Scherer, S.S.; Yum, S.W. Dominant Cx26 mutants associated with hearing loss have dominant-negative effects on wild type Cx26. *Mol Cell Neurosci.* **2011**, *47*, 71–78.

Zonta, F.; Girotto, G.; Buratto, D.; Crispino, G.; Morgan, A.; Abdulhadi, K.; Alkowari, M.; Badii, R.; Gasparini, P.; Mammano, F. The p.Cys169Tyr variant of connexin 26 is not a polymorphism. *Hum Mol Genet.* **2015**, *24*, 2641–2648.
